# The complete chloroplast genome sequences of three *Spondias* species reveal close relationship among the species

**DOI:** 10.1590/1678-4685-GMB-2017-0265

**Published:** 2019-03-11

**Authors:** Vanessa Santos, Cícero Almeida

**Affiliations:** 1 Universidade Federal de Alagoas Universidade Federal de Alagoas Laboratório de Recursos Genéticos Arapiraca Brazil Laboratório de Recursos Genéticos, Campus Arapiraca, Universidade Federal de Alagoas, Arapiraca, Brazil

**Keywords:** plastome, evolution, Spondias

## Abstract

This study reports the complete chloroplast sequences of three *Spondias* species. The genome sequences were obtained for *Spondias tuberosa*, *Spondias bahienses*, and *Spondias mombin* using the Illumina sequencing technology by a combination of *de novo* methods and a reference-guided assembly using *Sapindus mukorossi* as reference. The genomes of *S. tuberosa*, *S. bahiensis*, and *S. mombin* had 162,036, 162,218, and 162,302 bp, respectively. The coding regions exhibited 130 genes, including 34–35 tRNAs and 4 rRNAs. The results revealed synteny among the genomes, with high conservation in the gene order and content and CG content. The inverted repeat regions (IRA and IRB) and the large and small single copies were very similar among the three genomes. The phylogenomic analysis reported similar topologies as that of previous studies, which used partial chloroplast, wherein *S. mombin* was the first diverging lineage, while *S. tuberosa* and *S. bahiensis* were derived, indicating that the phylogenetic analysis using partial or complete genome produces similar results. In summary, (1) we presented the first complete chloroplast genome for the genus *Spondias*, (2) phylogenies analyzed using the complete chloroplast genomes revealed a robust phylogenetic topology for *Spondias*, and (3) gene order, content, and orientation in *Spondias* are highly conserved.

## Introduction

The genus *Spondias*, belonging to the family Anacardiaceae (order: Sapindales), comprises about 20 species and is of economic, ecological, and social importance, as certain species are utilized in agriculture and industry for human and animal food. Some species are occur in Brazil, such as *Spondias mombin* Jacq, *Spondias purpurea* L., *Spondias tuberosa* Arruda Camara, *Spondias venulosa* Mart. ex Engl, *Spondias bahiensis* P. Carvalho, van den Berg & M. Machado, and *Spondias dulcis* Parkinson (Mitchell and Daly, 2015). These species have 2n = 32 chromosomes with similar chromosome morphology among the *Spondias* species (Almeida *et al.*, 2007). Recent phylogenetic analysis has indicated that these species are closely related, wherein the species *S. tuberosa*, *S. venulosa*, and *S. bahiensis* are the most derived ones, and *S. purpurea*, *S. mombin*, and *S. dulcis* belong to a basal clade (Machado *et al.*, 2015; [Bibr B22] Silva *et al.*, 2015). However, these species are neglected in genetic studies, and only few genomic studies exist for the genus. In this context, sequencing the complete chloroplast genome is essential for studying the phylogeny and evolution of the genus, family Anacardiaceae, and order Sapindales. The complete chloroplast genome has been utilized for phylogenetic analysis with robust branch support in other taxa (Barret *et al., 2016*).

Chloroplasts are essential components of plants and are important for photosynthesis, biosynthesis, and carbon sequestration. These cytoplasmic organelles have a genome independent of the nuclear genome, which is inherited through the female plant only. Their genome is organized in a circular structure with a 100–200 kbp size range and a “quadripartite” structure comprising two large inverted repeats (IRs), which include the ribosomal genes and other plastid genes, separated by a large single copy (LSC) region and a small single copy (SSC) region. In general, the chloroplasts comprise 16S, 23S, 5S, and 27–31 tRNA genes, which are sufficient to translate all the amino acids, including three genes for the RNA polymerase subunit (similar to prokaryotes) and a majority of the genes for photosystem I, photosystem II, cytochrome, and ATP synthesis (revised by Green *et al.*, 2011), totaling approximately 80 proteins (Huang *et al.*, 2013). Plastid genomes are widely used for studies in taxonomy, phylogeny, phylogeography, and molecular identification of plants using *rbc*L and *mat*K genes and the intergenic *trn*H–*psb*A spacer as DNA bar coding (Hollingsworth *et al.*, 2009). As the chloroplast genomes are haploid and highly conserved regarding the genetic content and genomic structure, they have been widely used to study the evolutionary relationships of different taxonomic levels in plants. For *Spondias*, only two genes and one intergenic spacer have been used in the phylogenetic analyses, and the complete genome sequencing has a potential to resolve the hybrid origin for certain species.

With the advent of next-generation DNA sequencing technologies, there has been an increase in the number of chloroplast genomes sequenced; however, within the order Sapindales, which comprises 460 genera and 5670 species, only few plastid genomes have been sequenced. This study aimed to sequence the plastid genomes of *S. tuberosa*, *S. bahiensis*, *S. mombin* using high-throughput sequencing technology and to construct a phylogeny utilizing the complete chloroplast genomes of *Spondias*.

## Material and Methods

### Plant material, DNA isolation, and high-throughput DNA sequencing

*Spondias* plant material was collected in the state of Alagoas, Brazil, and total DNA was extracted (including nuclear, chloroplast, and mitochondrial DNA) using approximately 2 cm^2^ of leaves following the cetyltrimethylammonium bromide (CTAB) extraction method (Doyle and Doyle, 1987). The quality and quantity of the extracted DNA were verified by visualization on 1% agarose gel and spectrophotometry, respectively. The DNA sample was fragmented into pieces of 400–500 bp to construct the sequencing library. The fragments were ligated with adapters using the Nextera DNA Sample Preparation kit (Illumina), and 100 nt single-end reads for *S. bahiensis* and 100 nt paired-end reads for *S. tuberosa* and *S. mombin* were obtained by Illumina HiSeq2500 sequencing, done at the Central Laboratory for High Performance Technologies in Life Sciences (LaCTAD-*Laboratório Central de Tecnologias de Alto Desempenho em Ciências da Vida*) at the State University of Campinas (UNICAMP, Campinas, SP, Brazil).

### Chloroplast genome assembly and annotation

To generate the genomes, four million reads were mapped to the plastid genome of *Sapindus mukorossi* as reference (Table 1), using the software Geneious 9.1 (http://www.geneious.com) and the Map-to-Reference tool with a minimum 85% of identity, including 15% for gaps. The draft genomes were corrected using *de novo* contigs obtained from 165 million reads for *S. tuberosa*, 77 million reads for *S. bahiensis*, and 23 million reads for *S. mombin*, by means of the Ray software (Boisvert *et al.*, 2012), with a minimum size of 500 nt and 8× coverage. The *de novo* contigs were then mapped using the draft genome as the reference, and the regions with gaps or errors were manually corrected.

**Table 1:** Chloroplast genomes sequenced in this study, and others utilized as reference or out-group in phylogenetic analysis.

Taxon	GenBank	References
*Spondias tuberosa*	KU756562	This study
*Spodnias bahiensis*	KU756561	This study
*Spondias mombin*	KY828469	This study
*Pistacia vera*	KY549635	unpublished
*Sapindus mukorossi*	KM454982	Yang *et al*., 2016
*Mangifera indica*	KY635882	Rabah *et al*., 2017
*Anacardium occidentale*	KY635877	Rabah *et al*., 2017
*Rhus chinensis*	KX447140	Lee *et al*., 2016
*Boswellia sacra*	KU756561	Khan *et al*., 2017

Validation was achieved by Sanger sequencing of the *trn*H–*psb*A, *mat*K, *trn*D–*trn*T, *acc*D–*psa*I, *rbl*C, and *trn*K–*rpd*16 regions, using primers described by Scarcelli *et al.* (2011). PCR analyses were performed using a volume of 50 μL, containing 5 μL of a reaction buffer, 1.5 mM MgCl_2_, 0.2 mM dNTPs, 1 U Taq DNA polymerase, 0.5 μM of each primer, and 200 ng of DNA. Amplification was achieved with an initial denaturation at 94 °C for 3 min, followed by 40 cycles at 94 °C for 30 s, annealing at 55–60 °C for 30 s, and a final extension at 72 °C for 10 min. The PCR experiments were performed in a BioCycler thermocycler (Thermo Fisher Scientific), and the PCR products were subjected to electrophoresis on 1% agarose gels to confirm the amplification. The PCR products were then sequenced using BigDye^®^ Terminator v3.1 Cycle Sequencing Kit (Applied Biosystems^®^) on a 3500 Genetic Analyzer (Applied Biosystems Inc., Foster City, CA, USA).

Genome annotation was achieved by the Geneious software, using *S. mukorossi* as the reference, and it was checked with the annotation achieved using Verdant (Mckain *et al.*, 2016). For the annotation using Geneious, a minimum of 80% identity cutoff between the genomes was considered. The annotations were individually checked, and if necessary, were manually corrected for start and stop codons. A graphic representation of the plastomes was created using Organellar Genome DRAW (Lohse *et al.*, 2013).

### Genome comparison and phylogenetic analyses

The chloroplast genome sequences were aligned using the program MAFFT v7.017 (Katoh and Standley, 2013) implemented as the Multiple align tool in Geneious R9. The GTR model was determined using the Bayesian Information Criterion Evolutionary implemented in MEGA7 software ([Bibr B14] Kumar *et al.*, 2016). The evolutionary history was inferred by using the Maximum Likelihood method, and branch support was assessed with 1000 bootstrap replicates conducted in MEGA7 software. The three Spondias chloroplast genomes were compared using a BLAST Ring Image Generator assuming the default software settings (Alikhan *et al.*, 2011), and the number of interspecific SNPs were identified using Geneious. Single sequence repeats (SSRs) or microsatellites were identified using Phobos software (Mayer, 2010).

## Results

### Genome assembly and validation

The Illumina single-end and paired-end reads from *Spondias* species were mapped to the *S. mukorossi* chloroplast genome to obtain the draft genomes. The draft genomes revealed few errors and/or mapping failures of the reads (approximately 1–2% of the genomes), which were corrected using *de novo* contigs. After obtaining the final genomes, the reads were mapped with no errors and 100% identity, resulting in an average coverage of 150×. In addition, the *trn*H–*psb*A, *mat*K, *trn*D–*trn*T, *acc*D–*psa*I, *rbl*C, and *trn*K–*rpd*16 regions were sequenced using the Sanger method and aligned with the genome, resulting in high identity as the regions indicated intraspecific variation.

### Comparative chloroplast genome analysis

**Figure 1:**
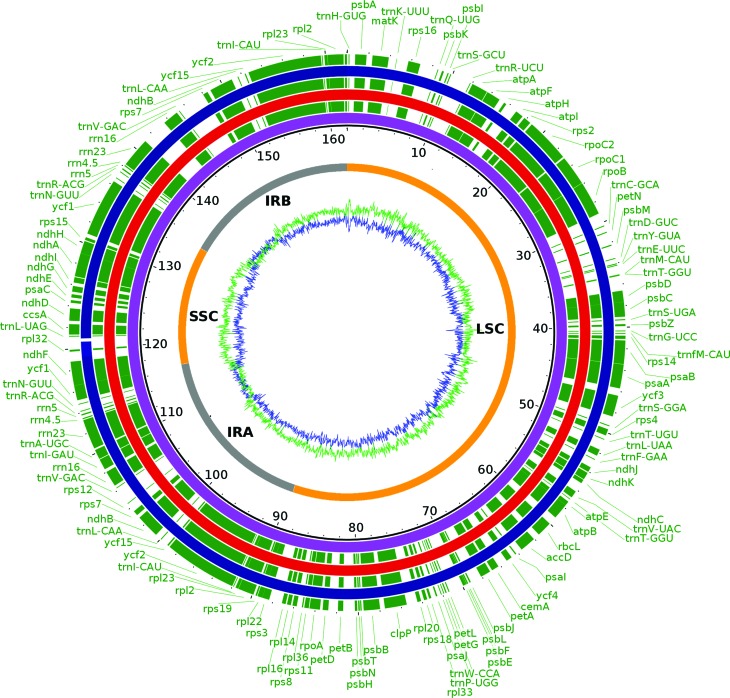
Complete gene map of *Spondias* chloroplast genomes. Gene annotations are represented in green. The chloroplast genomes are represented in purple (*S. tuberosa*), red (*S. bahiensis*), and blue (*S. mombin*). LSC: large single copy region; SSC: small single copy region; IR: inverted repeat. The green ring represents the A+T contents and the blue ring indicates C+G contents. The numbers near to *S. tuberosa* (purple circle) represent the nucleotide positions (in kbp).

The plastid genome sizes of *S. tuberosa, S. bahiensis,* and *S. mombin* were 162,039, 162,218, and 162,302 bp, respectively (Table 2). All the genomes indicated a conserved structure with a pair of inverted repeats, IRA and IRB, separated by the LSC and SSC regions (Figures S1, S2, and S3). For the *Spondias* species, the CG content was 37.6–37.7% and the length of the IR, LSC, and SSC regions were almost identical, revealing high similarity among the genomes (Table 2). The genomes comprised 130 genes, including 34–35 *t*RNA*s* and 4 *r*RNAs (Tables 2 and 3), and the arrangements of these regions were exclusively collinear (Figure 1). All protein-coding genes displayed AUG as the start codon; the coding sequences accounted for 63.8–64.2%, and the remaining regions included noncoding sequences (intergenic spacers).

**Table 2:** Summary of the chloroplast genome characteristics within the Anacardiaceae family. Genome size (bp), GC content (%), large single copy region - LSC (bp), small single copy region - SSC (bp), inverted repeat - IR (bp), N. of protein-coding genes, N. of tRNAs, and N. of rRNAs.

Species	Characteristics							
	Size (bp)	GC (%)	LSC	SSC	IR	Genes	tRNAs	rRNAs
*Spondias tuberosa*	162,039	37.7	89,453	18,369	27,139	130	35	4
*Spondias bahiense*	162,218	37.7	89,606	18,381	27,156	130	34	4
*Spondias mombin*	162,302	37.6	89,938	18,094	27,135	130	35	4
*Pistacia vera*	160,674	37.9	88,236	19,086	26,676	126	37	4

**Table 3:** List of genes present in the *Spondias tuberosa* chloroplast genome, obtained by genome annotation using *Sapindus mukorossi* as reference.

	Group of genes	Name of genes
**Protein synthesis and DNA replication**	Transfer RNAs	trnA-UGC (2x), trnC-GCA, trnD-GUC, trnE-UUC, trnF-GAA, trnfM-CAU, trnG-UCC, trnH-GUG, trnI-CAU (2x), trnI-GAU (3x) trnK-UUU, trnL-CAA(2x), trnL-UAA, trnL-UAG, trnM-CAU, trnN-GUU (2x), trnP-UGG, trnQ-UUG, trnR-ACG (2x), trnR-UCU, trnS-GCU, trnS-GGA, trnS-UGA, trnT-GGU (2x), trnT-UGU, trnV-GAC (2x), trnV-UAC, trnW-CCA, trnY-GUA
	Ribossomal RNAs (16S, 23S, 4.5S, 5S)	rrn16 (2x), rrn23 (2x), rrn4.5 (2x), rrn5 (2x)
	Ribossomal Protein small subunit	rps2, rps3, rps4, rps7 (2x), rps8, rps11, rps12, rps14, rps15, rps16, rps18, rps19 (2x)
	Ribossomal Protein large subunit	rpl2 (2x), rpl14, rpl16, rpl20, rpl22, rpl23 (2x), rpl32, rpl33, rpl36
	Subunits (α, β, β‘, β“) of the DNA-dependent RNA polymerase	rpoA, rpoB, rpoC1, rpoC2
**Photosynthesis**	Photosystem I	psaA, psaB, psaC, psaI, psaJ
	Photosystem II	psbA, psbB, psbC, psbD, psbE, psbF, psbH, psbI, psbJ, psbK, psbL, psbM, psbN, psbT, psbZ
	Cythochrome b/f complex	petA, petB, petD, petG, petL, petN
	ATP synthase	atpA, atpB, atpE, atpF, atpH, atpI
	NADH-dehydrogenase	ndhA, ndhB (2×), ndhC, ndhD, ndhE, ndhF, ndhG, ndhH, ndhI, ndhJ, ndhK
	Large subunit RUBISCO	rbcL
**Miscellaneous**	Acetyl-CoA carboxylase	accD
	Cythochrome c biogenesis	ccsA
	Maturase	matK
	ATP-dependent protease	clpP
	Inner membrane protein	cemA
**Pseudogene unknown function**	Conserved hypothetical chloroplast ORFs	ycf1 (2×), ycf2 (2×), ycf3, ycf4, ycf15 (2x)

**Figure 2:**
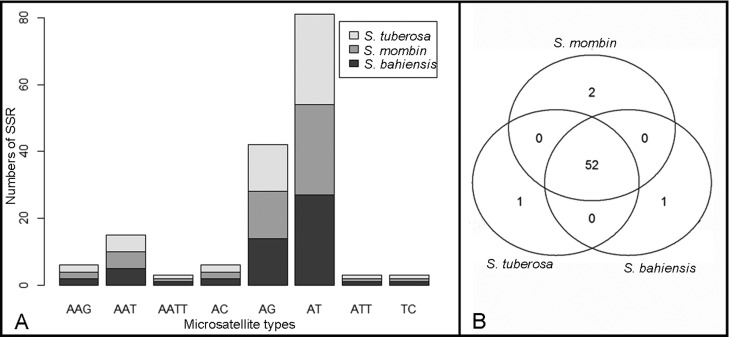
Comparative analysis of microsatellites in the chloroplast genomes of *Spondias*. (A) Microsatellite type distribution in three *Spondias* species. (B) Venn diagram showing the number of SSR that are shared among *S. bahiensis*, *S. tuberosa*, and *S. mombin*.

**Figure 3:**
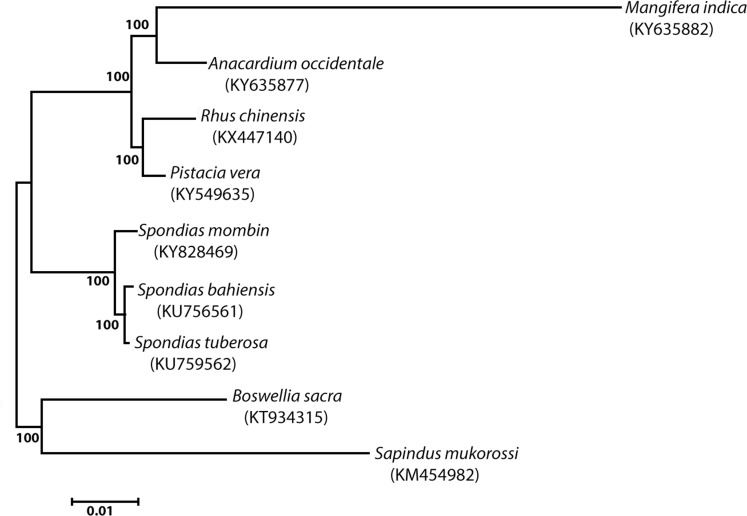
Molecular phylogenetic analysis by maximum likelihood method, with supported values estimated by bootstrap.

In the three genomes of the genus *Spondias* we found 159 SSRs, which were evenly distributed (Figure 2A), and the motifs AT and AG were more abundant. The *Spondias* species indicated similar SSR distribution, wherein *S. tuberosa* and *S. bahiensis* had one species-specific SSR each, and *S. mombin* had two species-specific SSRs (Figure 2B). The comparison among *Spondias* species indicated 856 SNPs between *S. tuberosa* and *S. bahiensis*, 3044 SNPs between *S. tuberosa* and *S. mombin*, and 3289 SNPs between S*. bahiensis* and *S. mombin*. The phylogenetic analysis revealed three clades, of which one clade was formed by *S. tuberosa* and *S. bahiensis*, the second clade by *S. mombin*, and the outgroup clade was formed by other species (Figure 3).

## Discussion

The assembly of the chloroplast genomes using single- or paired-end reads resulted in genomes with few errors due to the differences between the reads of the species and the reference genome utilized; however, these errors were easily identified and corrected by the alignment of the *de novo* contigs. Genetic analysis of the genus *Spondias* was performed using molecular (Machado *et al.*, 2015; Silva *et al.*, 2015) and cytogenetic data (Almeida *et al.*, 2007). These studies have utilized the partial chloroplast regions or expressed sequences for the phylogenetic analysis; however, the advent of next-generation sequencing technologies has increased the number of complete chloroplast genomes sequencing, allowing robust phylogenetic analysis. In this context, the complete chloroplast genome sequences are important to assess the phylogenetic relationships. The chloroplast genomes have been utilized for phylogenetic analyses in Arecaceae and they provide robust branch support for deep phylogenetic relationships among tribes of the subfamily (Barrett *et al.*, 2016).

The chloroplast genomes of the genus *Spondias* are highly similar, with only one deletion of tRNA in *S. bahiensis*. The order and structure of the IR, LSC, and SSC regions were exclusively collinear. The genome size, CG content, and the length of the IR, SLC, and SSC regions were almost identical, revealing high similarity among the genomes and suggesting low diversity within the genus *Spondias*. Remarkably, the chloroplast genomes of *S. tuberosa* and *S. bahiensis* are highly similar when compared with *S. mombin*, suggesting a close genetic relationship and indicating a possible hybrid origin for *S. bahiensis*. *S. bahiensis* has been described as a hybrid between *S. tuberosa* and *S. mombin* (Almeida *et al.*, 2007). Studies using chromosome banding and genomic *in situ* hybridization could not determine if *S. bahiensis* is a hybrid (Almeida *et al.*, 2007); however, molecular studies using phylogeny with chloroplast regions (Machado *et al.*, 2015; Silva *et al.*, 2015) and ESTs (Machado *et al.*, 2015) suggest that *S. bahiensis* is a new species and a hybrid between *S. tuberosa* and *S. venulosa*. In the present study, the comparison between *S. tuberosa* and *S. bahiensis* suggests the hybrid origin of *S. bahiensis*, with *S. tuberosa* as the female genitor; the SNP comparison displayed few SNPs between *S. bahiensis* and *S. tuberosa,* whereas *S. bahiensis* and *S. mombin* revealed several SNPs. The SSRs revealed high conservation in the three species, indicating low evolution of SSRs in the genus.

We demonstrate that the gene order, content, and orientation in *Spondias* are highly conserved, and that this observation is similar to other taxa, such as in *Aconitum* (Ranunculaceae) ([Bibr B19] Park *et al.*, 2017), *Salix* (Salicaceae) ([Bibr B11]Huang *et al.*, 2017), and in the gymnosperm genus *Pinus* (Asaf *et al.*, 2018), indicating that the chloroplast genomes reveal high synteny in close species; however, when analyzed across distant species of the Malpighiales order, three inversions were found in the LSC region (Cauz-Santos *et al.*, 2017). In summary, (1) we presented the first complete chloroplast genome sequences for the genus *Spondias*; (2) phylogenies analyzed using the complete chloroplast genomes revealed a robust phylogenetic topology for *Spondias*; and (3) gene order, content, and orientation in *Spondias* are highly conserved.

## Supplementary material

The following online material is available for this article:

Figure S1 Chloroplast genome map of *S. tuberosa*.

Figure S2 Chloroplast genome map of *S. bahiensis*.

Figure S3 Chloroplast genome map of *S. mombin*.
