# Supplementary material for: The complete chloroplast genome sequences of three Spondias species reveal close relationship among the species
Source: Genet Mol Biol. 2019 Mar 11;42(1):132–8. doi: 10.1590/1678-4685-GMB-2017-0265 (PMC6428118; doi:10.1590/1678-4685-GMB-2017-0265)
Supplement: Supplementary file 2 [file 1415-4757-GMB-1678-4685-GMB-2017-0265-20170265-suppl2.pdf]

Supplementary Material to "The complete chloroplast genome sequences of three *Spondias* species reveal close relationship among the species"

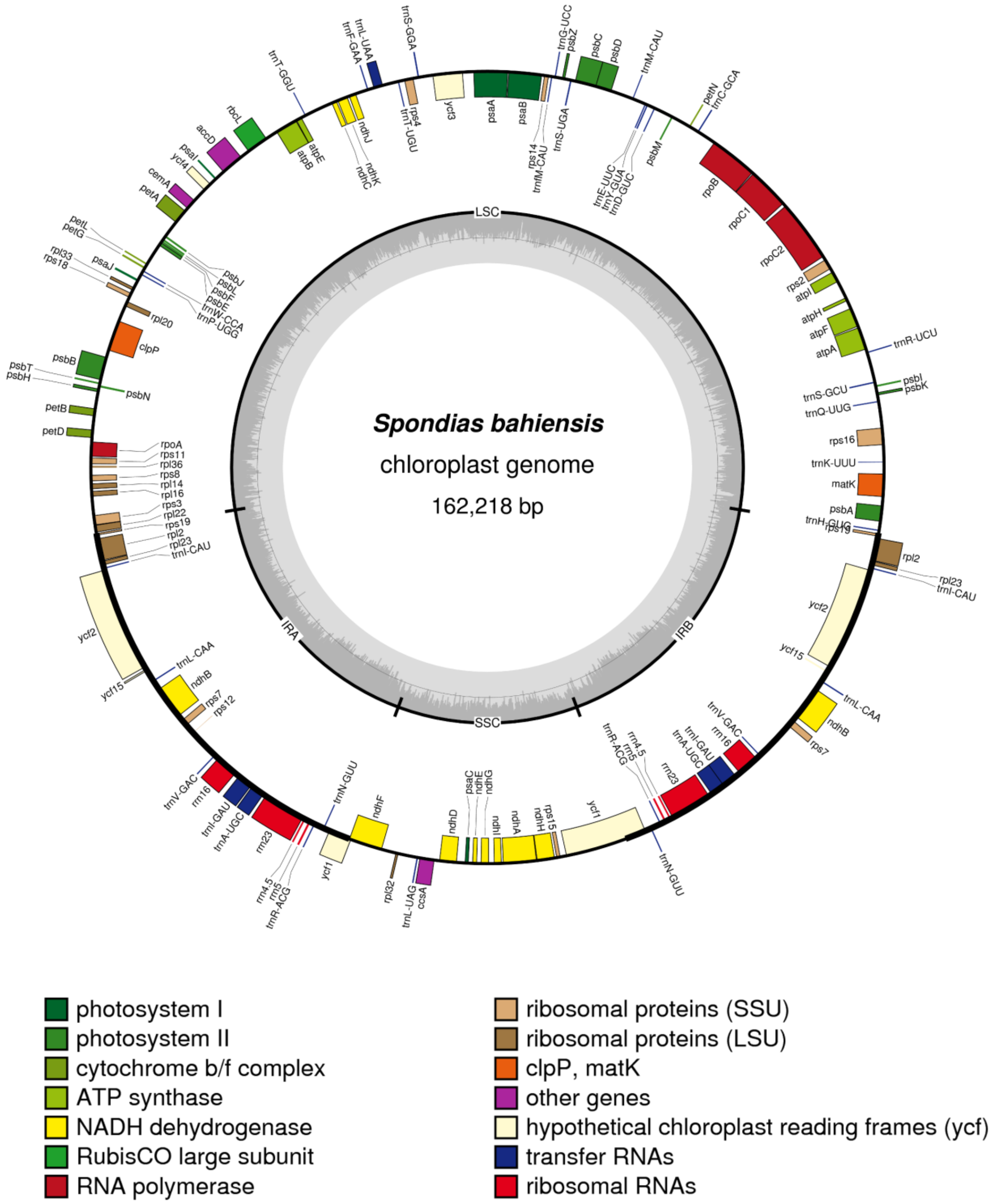

Figure S2 - Chloroplast genome map of *S. bahiensis*. Annotated genes are colored according to the functional categories as described in the legend. Genes on the inside are transcribed in a clockwise direction and genes in the outside are transcribed in a counterclockwise direction. LSC: large single copy region; SSC: small single copy region; IRA; IRB: inverted repeats. The inside gray circle represents the C+G content.
